# Translation, cultural adaptation and validation of the SCHNOS in French

**DOI:** 10.1186/s40463-019-0339-6

**Published:** 2019-03-20

**Authors:** Marie-Renée Atallah, Daniel Milad, Youcef-Hamza Benamer, Mikhail Saltychev, Sam P. Most, Sami P. Moubayed

**Affiliations:** 1 Division of Otolaryngology-Head and Neck Surgery, Department of Surgery, Université de Montréal, Montréal, Québec Canada; 2 Department of Physical and Rehabilitation Medicine, Turku University Hospital and University of Turku, Turku, Finland; 3 Division of Facial Plastic and Reconstructive Surgery, Department of Otolaryngology-Head & Neck Surgery, Stanford University School of Medicine, Stanford, CA USA

**Keywords:** Rhinoplasty, Nasal valve, Nasal obstruction, Surgery outcome, Quality of life, Instrument, Validation

## Abstract

**Background:**

The Standardized Cosmesis and Health Nasal Outcomes Survey (SCHNOS) is a validated questionnaire that assesses functional and aesthetic outcomes of rhinoplasty patients. There are 274 million French speakers worldwide, and this questionnaire is currently not available in French. The purpose of this study was to translate, adapt, and validate a French version of the SCHNOS questionnaire.

**Methods:**

The SCHNOS questionnaire was translated from English to French according to international guidelines. Ten French-speaking rhinoplasty patients were interviewed in order to evaluate the understandability and acceptability of the translation and produce a final version. The final version was administered prospectively to 25 rhinoplasty patients and 25 controls at two-week intervals. It was then administered to 165 consecutive patients. Psychometric properties were evaluated using the Item Reponse Theory (IRT) and confirmatory factor analysis (CFA).

**Results:**

Three items from the original SCHNOS were modified to produce the French-SCHNOS (F-SCHNOS). Discrimination abilities of F-SCHNOS-O and F-SCHNOS-C were perfect, with values of 2.18(*p* < 0.001, 95% CI 1.74 to 2.62) for SCHNOS-O and 2.62(*p* < 0.001, 95% CI 2.03 to 3.21). Internal consistency was high, with Cronbach’s alpha of 0.93 for F-SCHNOS-O and 0.95 for F-SCHNOS-C. IRT showed good psychometric properties with almost each step up or down across the scale associating with meaningful differences in outcome severity. All four SCHNOS-O items were equally “important” in defining the total score. The F-SCHNOS-C total score was defined by mostly four out of six items.

**Conclusions:**

The SCHNOS was translated, adapted, and psychometrically validated for use in a French-speaking population.

## Background

Rhinoplasty is one of the most common procedures in plastic surgery and otolaryngology [1]. The indications can be functional or cosmetic, and it is essential to evaluate both components, as cosmetic nasal surgery can have functional consequences, and vice-versa. The Standardized Cosmesis and Health Nasal Outcomes Survey (SCHNOS) is the first patient-reported outcome measure (PROM) developed using accepted international standards to evaluate both functional and cosmetic components of rhinoplasty [2].

French is one of the six official languages of the United Nations, and there are currently 274 million French speakers worldwide [3]. However, there is currently no available translation of the SCHNOS questionnaire in French. To ensure semantic and conceptual equivalence, it is recommended for PROMs to be culturally adapted when administered in a population with a different language and culture [4, 5]. This procedure allows not only for the evaluation of patients within their own cultural context, but also produces standardized instruments for comparisons among international groups of individuals [4].

The aim of this study was to carry out the translation and cultural adaptation of the SCHNOS questionnaire. The questionnaire was then validated with functional and/or cosmetic rhinoplasty patients in a French-speaking population using item-response theory (IRT) and confirmatory factor analysis (CFA).

## Methods

**Fig. 1:**
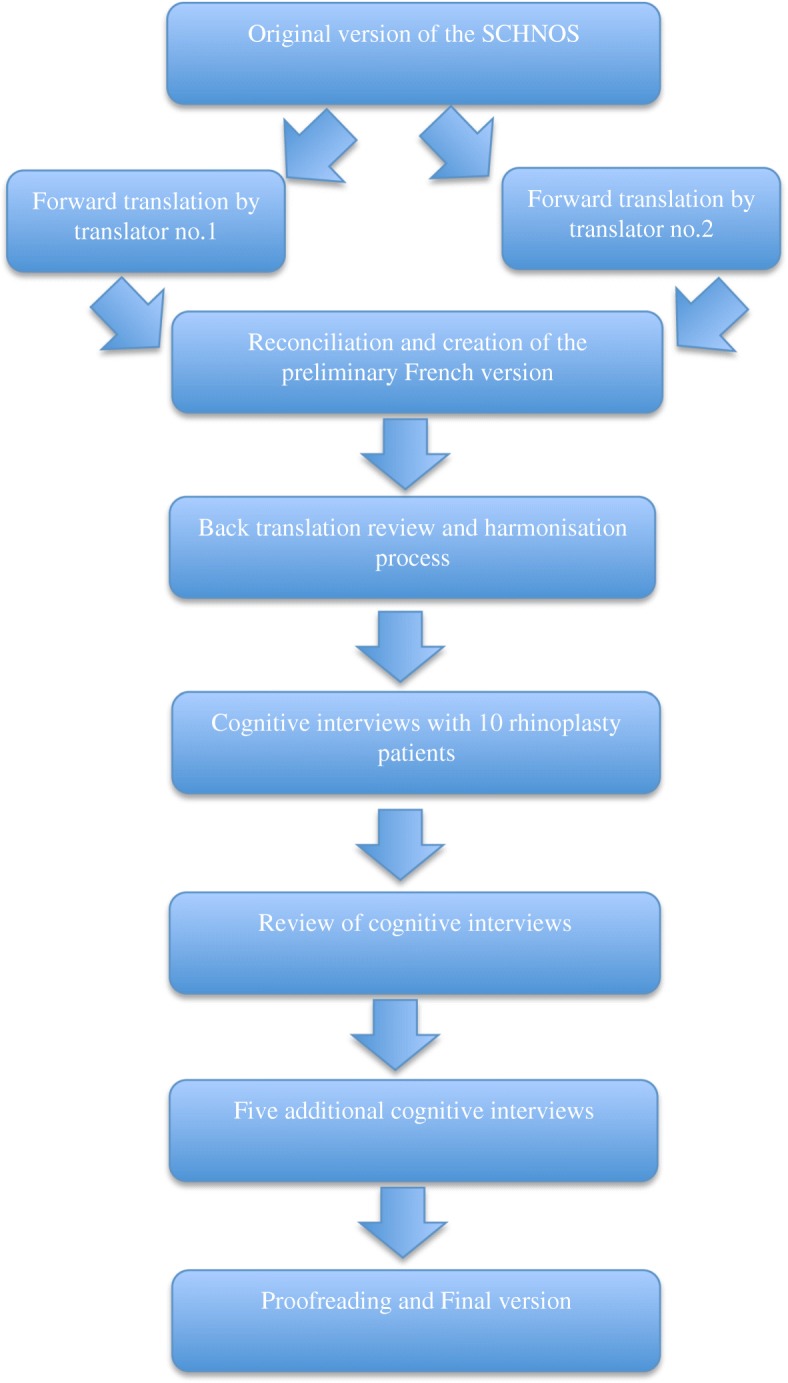
Translation process

The translation process and cultural adaptation was conducted with respect of the International Society for Pharmacoeconomics and Outcomes Research (ISPOR) guidelines [5] (Fig. 1). This prospective validation study was conducted in two phases. The questionnaire was first translated and cross-cultural adaptation was carried out. The second phase consisted in the psychometric validation of the questionnaire. The protocol was approved by our institutional review board.

### Questionnaire description

The SCHNOS is a 10-item self-rated questionnaire that uses a Likert-like 0–5 scale (‘no problem’ to ‘extreme problem’). The SCHNOS does not produce a combined total score, but two scores – one for each domain, an obstruction score (SCHNOS-O) and a cosmesis score (SCHNOS-C). The SCHNOS-O is calculated as a sum of scores of items 1–4 divided by 20 and multiplied by 100. The SCHNOS-C score is calculated as a sum of scores of items 5–10 divided by 30 and multiplied by 100.

### Translation process

Standard forward and back-translation procedure was followed. Two independent translators, both native speakers of the target language (French) and fluent in English, translated the SCHNOS questionnaire from English to French. The first and senior authors then reconciled and merged the two initial versions of each questionnaire into a harmonized French version. The harmonized version was then back-translated into English by a third independent native English-speaking translator that was unaware of the original version of the questionnaire. This back-translation was reviewed and compared with the original version to ensure that the initial concepts were respected and to identify discrepancies with the original questionnaires. Interviews were then conducted with ten rhinoplasty patients that were native French speakers. Informed written consent was obtained for all patients. During the 15-min interviews, the first author, a native French speaker and fluent in English, reviewed each questionnaire with the patients to identify ambiguities and to verify its understandability and acceptability. Patients were then asked to verbalize their perception of each item in the three questionnaires. Written notes were taken during the interviews to compile the answers. Data gathered was reviewed and modifications were made to the translated versions. Five additional interviews were conducted to review those modifications. We then performed a final proofreading and a final version of was elaborated.

### Test-retest

The final French version of the SCHNOS (F-SCHNOS) was administered, on two occasions, to 25 adult rhinoplasty patients and 25 controls in a tertiary Facial Plastic Surgery clinic. Exclusions criteria included rhinoplasty that was performed less than a month ago, concomitant endoscopic sinus surgery, and inability to understand written or oral French. The control group consisted of adult patients presenting for a chief complaint that was neither nasal deformity nor nasal obstruction. Informed written consent was obtained and each questionnaire was filled out on the day of consultation. Patients were contacted by phone 2 weeks later to complete the questionnaire. Spearman’s rank correlation and a Wilcoxon signed-rank test for matched pairs (*p*-values) we used to compare the scores obtained for F-SCHNOS-O and F-SCHNOS-C during the 2 weeks interval.

### Psychometric validation

One hundred and sixty-five consecutive patients presenting a Facial Plastic Surgery clinic completed the F-SCHNOS, as part of routine clinical data collection. This data was then retrospectively collected after institutional review board approval.

Internal consistency was defined by a Cronbach’s alpha reported along with its one-sided (lower) 95% confidence limit (95% CL). Alpha > = 0.9 was considered excellent, 0.9 > alpha> = 0.8 good, 0.8 > alpha> = 0.7 acceptable, 0.7 > alpha> = 0.6 questionable, 0.6 > alpha> = 0.5 poor, and < 0.5 unacceptable.

In general, the IRT analysis defines discrimination and difficulty parameters of a questionnaire. A discrimination parameter describes the sensitivity of the test to differentiate different severity levels of symptoms. The steeper the regression curve, the more discriminative the test is. In this study, discrimination of 0.01 to 0.24 was considered ‘none’ (a totally level regression curve), 0.25 to 0.64 was considered ‘low’, 0.65 to 1.34 was considered ‘moderate’, 1.35 to 1.69 was considered ‘high’; and a discrimination ≥1.7 was considered ‘perfect’ (a regression curve approaching a vertical line) [6]. Ideally, the steepest interval corresponds to the patients who obtained average F-SCHNOS-O or F-SCHNOS-C total scores in the studied population. In turn, the difficulty parameter refers to the level of a perceived nasal problem needed to achieve a 50% probability of choosing a particular score. In an ideal situation, patients who experience an average nasal problem (in this particular population) should have a 0.5 probability of getting a score located at the middle of a scale. Thus, in this “best possible” example, the responses of ‘0’ or ‘1’ points in SCHNOS items would produce ‘difficulty estimates’ with a minus sign, indicating that the respondent perceived a lower level of symptoms than the average within the population. Respectively, estimates for responses with ‘4’ and ‘5’ points would carry a positive sign, indicating that the respondent perceived higher levels of symptoms than the average within this population. The IRT Rating Scale Model (RSM) was used. Item information and test characteristic curves and the test information functions were presented graphically. All the IRT and other analyses (except CFA) were performed using Stata/IC Statistical Software: Release 15, College Station (StataCorp LP, TX, USA).

The root mean square error of approximation (RMSEA) was used as a primary indicator of the goodness of model fit in CFA. The modification indices suggested by the software were used to impute covariances between factors one at a time, each time testing the RMSEA closeness to the value of ≤0.05 (the threshold for accepting the model fit). The relative chi-squared test was used to reduce dependence on the sample size with value < 3.0 pointed to an acceptable fit. The CFA was conducted using IBM® SPSS® Amos™, Version 25.0 (IBM® Corp. Released 2017, PA, USA).

All *p*-values was considered statistically significant if = < 0.05 when not mentioned otherwise.

## Results

### Translation and cultural adaptation

There were multiple differences between the two forward translations, which were reconciled and harmonized, as shown in Table 1. Back-translation showed only minimal discrepancies with the original concepts requiring no further modification. Ten interviews were conducted with two preoperative and eight postoperative rhinoplasty patients (four women, six men, mean age 39.4). Three modifications were made after cognitive interviews: [1] the word *du* in the introduction was underlined, [2] we added *(bout du nez)* at the end of item no. 6 to clarify pointe du nez, and [3] item no.7 was modified to add *(vu de face)* at the end of the sentence. These modifications were presented to five additional postoperative rhinoplasty patients (two women, three men). The final version and scoring of the final French version compared to the original English version is seen in Fig. 2.

**Table 1:** Reconciliation of forward translations

# Item	Original version (English)	Forward translation no.1	Forward translation no.2	Harmonized version	Rationale
2	Getting air through my nose during exercise	Respirer par le nez au cours de l’exercice physique	Difficulté à respirer par le nez pendant l’activité physique	Difficulté à respirer par le nez pendant l’exercice physique	Researchers chose the second translation, as it is more accurate in relation to the introduction statement. Adding *difficulté à* reflects more precisely the idea that “getting air through the nose during exercise” is a problem.
3	Having a congested nose	Avoir le nez congestionné	Nez congestionné	Nez congestionné	Researchers discussed that both translations had the same meaning, but the second translation was shorter and simpler
5	Decreased mood and self-esteem due to my nose	Mauvaises humeur et estime de soi à cause de mon nez	Perte d’humeur ou d’estime de moi à cause de mon nez	Mauvaises humeur et estime de soi à cause de mon nez	Researchers discussed that the first translation reflected more properly the original concept in the English version
7	The straightness of my nose	À quel point mon nez est. droit	La rectitude de mon nez	À quel point mon nez est. droit	The first translation was chosen, as it is a colloquial expression of more common usage in the French speaking population than the word “rectitude”.
9	How well my nose suits my face	Combien mon nez convient à mon visage	Comment mon nez s’agence avec mon visage	Comment mon nez s’agence avec mon visage	Researchers discussed that in French, the first translation was more of quantification and would reflect “how much” instead of “how well”. The second translation is a qualifier and thereby conceptually equivalent to the original version.

**Fig. 2:**
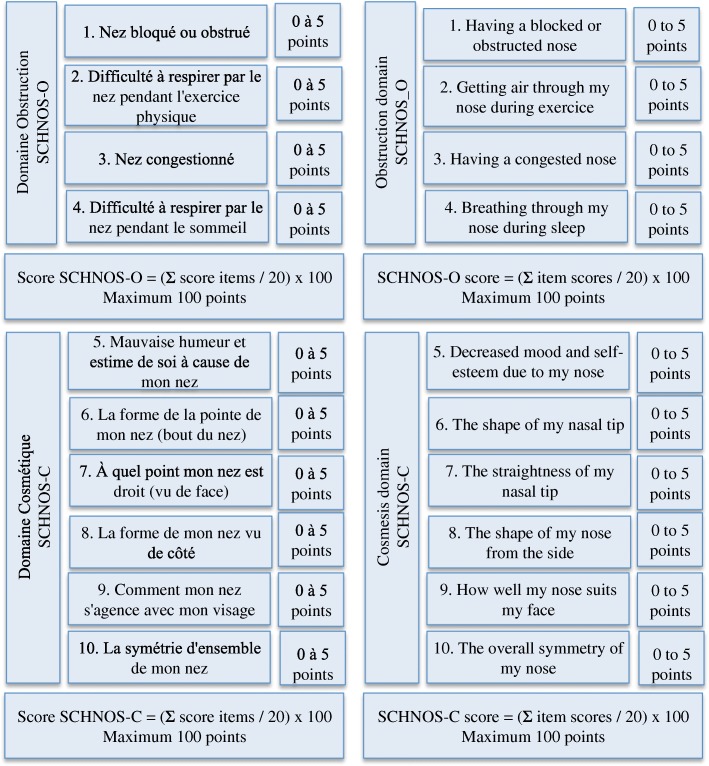
The structures and scoring formulas of English and French versions of SCHNOS

### Test-retest

Of the 52 patients invited to answer the questionnaires, 50 were recruited (96% response rate). The patient group consisted of 15 women and 11 men (mean age 40.1 years) presenting for preoperative (*n* = 12) or postoperative (*n* = 13) rhinoplasty appointments. The patient group consisted of 13 women and 12 men (mean age 49.9 years) presenting for a variety of non-rhinoplasty related reasons (Mohs’ reconstruction of non-nasal sites, facial paralysis, temporomandibular disorders, facial trauma, trigeminal neuralgia, among others). The spearman’s rank correlation for SCHNOS-O and SCHNOS-C were positive and statistically significant (SCHNOS-O: *r* = 0,606 on 50 observations (95% CI: 0,294 to 0,757); SCHNOS-C: *r* = 0,762 on 50 observations (95% CI: 0,614 to 0,859)). No statistical significance was found in responses obtained in the Wilcoxon signed-rank test for SCHNOS-C (*p* = 0,5920). There was a significant difference for the SCHNOS-O (*p* = 0,0060).

### Psychometric validation

**Fig. 3:**
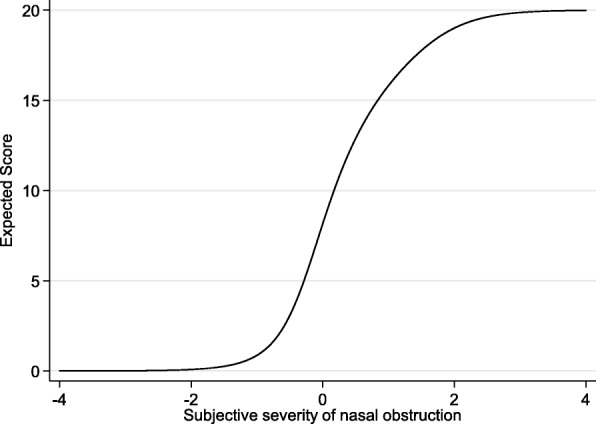
Test characteristic curve of F-SCHNOS-O

**Fig. 4:**
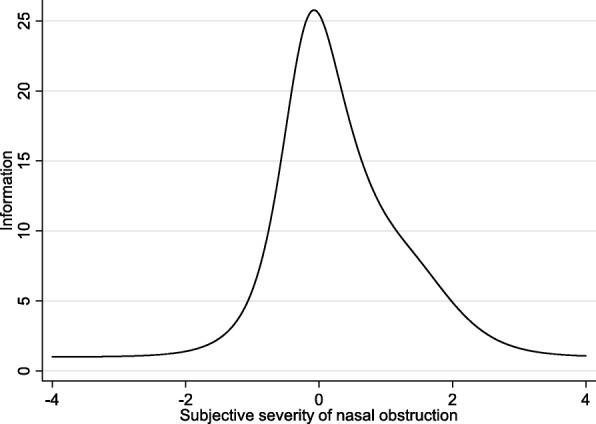
Test information function of F-SCHNOS-O

**Fig. 5:**
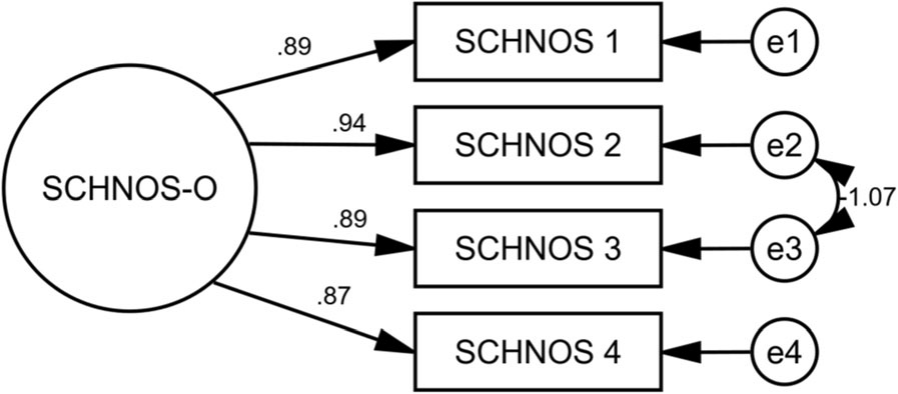
Path diagram of factor structure of F-SCHNOS-O. Circles represent unmeasured variables (in terms of factor analysis, ‘latent traits’, ‘constructs’, or ‘factors’), while square shapes represent measured observed variables (the SCHNOS items in this study). Factor loadings (relationships between observed variables and a factor/unmeasured variable) are marked as single-headed arrows. Correlations between two unobserved variables are shown as two-headed arrows. Conventionally, measurement errors are presented as smaller circles denoted as “e1”, “e2”, etc. Numbers in bold by the square shapes are coefficients of determination, displaying how much of the total variance of a latent factor is explained by the variance present in a particular observed variable. Other numbers in the diagram show the strengths of loadings and correlations

The results from IRT analysis are available from 165 individual patients (75 female, 89 male; 69 septorhinoplasty, 95 non-septorhinoplasty; mean age 48.6 years) and are shown in Table 2 for F-SCHNOS-O and Table 3 for F-SCHNOS-C. The internal consistency of F-SCHNOS-O was good with alpha 0.93 (lower 95% CL 0.92). The discrimination ability of F-SCHNOS-O was perfect 2.18 (*p* < 0.001, 95% CI 1.74 to 2.62). Even though some steps up or down across the SCHNOS-O scale were insignificant, most of them were associated with a meaningful differences in severity of the measured nasal problem perceived by a patient (Table 2). The difficulty estimates demonstrated an ideal distribution around the middle point of the F-SCHNOS-O scale – step 3 vs. 2. For the entire F-SCHNOS-O score, most information could be obtained around the average level of perceived problem (Figs. 3 and 4). The CFA model of F-SCHNOS-O did not achieve the level of statistical significance with RMSEA 0.08 (Table 4). However, it seems that all four items were equally “important” in defining the total SCHNOS-O score – correlations varied from 0.87 to 0.94 (Fig. 5). There was one imputed covariance between items #2 and #3.

**Table 2:** Difficulty parameters of F-SCHNOS-O item – Rating Scale Model RSM (*n* = 165)

Item	Step	Difficulty	*p*-value	95% CI	
SCHNOS 1	1 vs. 0	−0.27	0.020	−0.50	−0.04
	2 vs. 1	−0.37	0.003	−0.61	− 0.13
	3 vs. 2	0.04	0.749	−0.19	0.26
	4 vs. 3	0.43	< 0.001	0.22	0.65
	5 vs. 4	1.37	< 0.001	1.09	1.66
SCHNOS 2	1 vs. 0	−0.08	0.486	−0.30	0.14
	2 vs. 1	−0.18	0.131	−0.42	0.05
	3 vs. 2	0.23	0.047	0.00	0.45
	4 vs. 3	0.62	< 0.001	0.40	0.85
	5 vs. 4	1.56	< 0.001	1.26	1.87
SCHNOS 3	1 vs. 0	−0.26	0.022	−0.49	−0.04
	2 vs. 1	−0.37	0.003	−0.61	−0.13
	3 vs. 2	0.04	0.719	−0.18	0.26
	4 vs. 3	0.44	< 0.001	0.22	0.66
	5 vs. 4	1.38	< 0.001	1.09	1.66
SCHNOS 4	1 vs. 0	−0.17	0.146	−0.39	0.06
	2 vs. 1	−0.27	0.027	−0.51	−0.03
	3 vs. 2	0.14	0.220	−0.08	0.36
	4 vs. 3	0.54	< 0.001	0.32	0.76
	5 vs. 4	1.48	< 0.001	1.18	1.77

**Table 3:** Difficulty parameters of F-SCHNOS-C item – Rating Scale Model RSM (*n* = 163)

Item	Step	Difficulty	*p*-value	95% CI	
SCHNOS 5	1 vs. 0	0.67	< 0.001	0.48	0.86
	2 vs. 1	0.44	< 0.001	0.24	0.64
	3 vs. 2	0.63	< 0.001	0.43	0.83
	4 vs. 3	1.09	< 0.001	0.86	1.32
	5 vs. 4	1.21	< 0.001	0.96	1.46
SCHNOS 6	1 vs. 0	1.02	< 0.001	0.80	1.24
	2 vs. 1	0.79	< 0.001	0.58	1.00
	3 vs. 2	0.98	< 0.001	0.76	1.20
	4 vs. 3	1.44	< 0.001	1.17	1.72
	5 vs. 4	1.56	< 0.001	1.27	1.86
SCHNOS 7	1 vs. 0	0.73	< 0.001	0.54	0.92
	2 vs. 1	0.50	< 0.001	0.30	0.70
	3 vs. 2	0.69	< 0.001	0.48	0.89
	4 vs. 3	1.15	< 0.001	0.92	1.39
	5 vs. 4	1.27	< 0.001	1.01	1.53
SCHNOS 8	1 vs. 0	0.79	< 0.001	0.59	0.99
	2 vs. 1	0.56	< 0.001	0.35	0.76
	3 vs. 2	0.74	< 0.001	0.54	0.95
	4 vs. 3	1.21	< 0.001	0.97	1.45
	5 vs. 4	1.33	< 0.001	1.06	1.59
SCHNOS 9	1 vs. 0	0.88	< 0.001	0.68	1.09
	2 vs. 1	0.65	< 0.001	0.45	0.86
	3 vs. 2	0.84	< 0.001	0.63	1.05
	4 vs. 3	1.30	< 0.001	1.05	1.56
	5 vs. 4	1.42	< 0.001	1.15	1.70
SCHNOS 10	1 vs. 0	0.80	< 0.001	0.60	1.00
	2 vs. 1	0.57	< 0.001	0.36	0.77
	3 vs. 2	0.75	< 0.001	0.55	0.96
	4 vs. 3	1.22	< 0.001	0.98	1.46
	5 vs. 4	1.34	< 0.001	1.07	1.60

**Table 4:** Construct of F-SCHNOS-O

Method	Value			
Root mean squared residual (RMR)	0.03			
Goodness of fit index (GFI)	0.99			
Adjusted goodness of fit index (AGFI)	0.93			
Parsimony goodness of fit index (PGFI)	0.10			
Bentler-Bonett normed fit index (NFI)	1.00			
Bollen’s relative fit index (RFI)	0.98			
Bollen’s incremental fit index (IFI)	1.00			
Tucker-Lewis coefficient (TLI)	0.99			
Bentler’s comparative fit index (CFI)	1.00			
Parsimony ratio (PRATIO)	0.17			
Parsimony adjustment to the NFI (PNFI)	0.17			
Parsimony adjustment to the CFI (PCFI)	0.17			
Akaike information criterion (AIC)	20			
Browne-Cudeck criterion (BCC)	21			
Bayes information criterion (BIC)	47			
Bozdogan’s consistent AIC (CAIC)	56			
Hoelter’s ‘critical N’ for a significance level of .05 (HOELTER .05)	272			
Hoelter’s ‘critical N’ for a significance level of .01 (HOELTER .01)	469			
Method	Value	90% CI		
Noncentrality parameter (NCP)	1.0	0.0	9.4	
Minimum value of the discrepancy (FMIN)	0.01	0.0	0.07	
Root mean squared error of approximation (RMSEA)	0.08	0.0	0.26	
Except for a constant scale factor (ECVI)	0.14	0.13	0.2	
Method	Value	DF	*p*-value	CMIN/DF
Minimized value of the discrepancy function (CMIN)	2.0	1	0.16	2.0

Tests of the goodness of fit used. All figures are shown for the default model fit

**Fig. 6:**
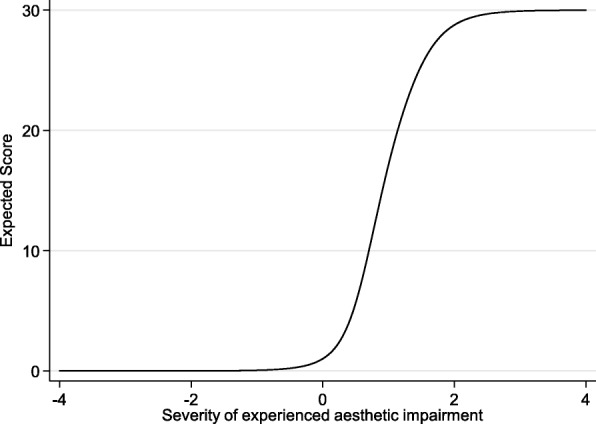
Test characteristic curve of F-SCHNOS-C

**Fig. 7:**
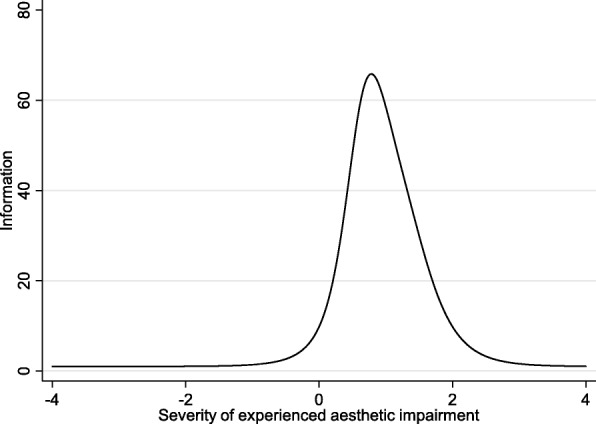
Test information function of F-SCHNOS-C

**Fig. 8:**
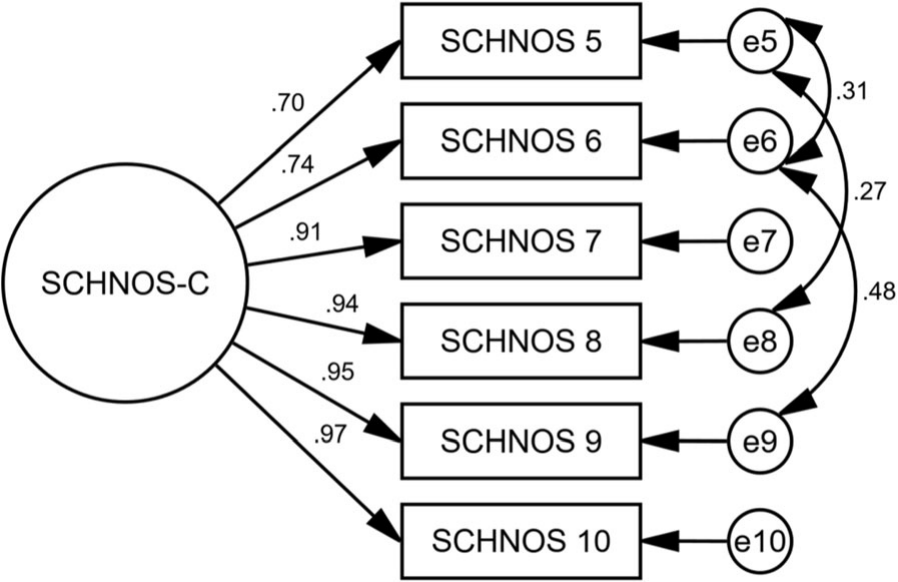
Path diagram of factor structure of F-SCHNOS-C. Circles represent unmeasured variables (in terms of factor analysis, ‘latent traits’, ‘constructs’, or ‘factors’), while square shapes represent measured observed variables (the SCHNOS items in this study). Factor loadings (relationships between observed variables and a factor/unmeasured variable) are marked as single-headed arrows. Correlations between two unobserved variables are shown as two-headed arrows. Conventionally, measurement errors are presented as smaller circles denoted as “e1”, “e2”, etc. Numbers in bold by the square shapes are coefficients of determination, displaying how much of the total variance of a latent factor is explained by the variance present in a particular observed variable. Other numbers in the diagram show the strengths of loadings and correlations

The internal consistency of F-SCHNOS-C was good – alpha 0.95 (lower 95% CL 0.94). The discrimination ability of F-SCHNOS-C was perfect – 2.62 (*p* < 0.001, 95% CI 2.03 to 3.21). All the steps up or down across the F-SCHNOS-C scale were associated with a meaningful difference in severity of the measured nasal problem perceived by a patient (Table 3). When analyzing the difficulty parameter for F-SCHNOS-C items, the scale tended to underestimate the severity of cosmetic disadvantage experienced by patients. In other words, a patient who has placed a mark on the lower end of the scale might experience more cosmetic disadvantage than on average in the studied population. Respectively, the information curve of F-SCHNOS-C was shifted towards greater severity of symptoms (Figs. 6 and 7). The CFA model for F-SCHNOS-C did not achieve the level of clinical significance – RMSEA 0.08 (Table 5). However, it seems that items #7, #8, #9 and #10 define the total F-SCHNOS-C score much more than items #5 and #6 (Fig. 8). Three covariances needed to be added to the model.

**Table 5:** Construct of SCHNOS-C

Method	Value			
Root mean squared residual (RMR)	0.05			
Goodness of fit index (GFI)	0.97			
Adjusted goodness of fit index (AGFI)	0.90			
Parsimony goodness of fit index (PGFI)	0.28			
Bentler-Bonett normed fit index (NFI)	0.99			
Bollen’s relative fit index (RFI)	0.97			
Bollen’s incremental fit index (IFI)	0.99			
Tucker-Lewis coefficient (TLI)	0.99			
Bentler’s comparative fit index (CFI)	0.99			
Parsimony ratio (PRATIO)	0.40			
Parsimony adjustment to the NFI (PNFI)	0.40			
Parsimony adjustment to the CFI (PCFI)	0.40			
Akaike information criterion (AIC)	42			
Browne-Cudeck criterion (BCC)	44			
Bayes information criterion (BIC)	86			
Bozdogan’s consistent AIC (CAIC)	101			
Hoelter’s ‘critical N’ for a significance level of .05 (HOELTER .05)	150			
Hoelter’s ‘critical N’ for a significance level of .01 (HOELTER .01)	200			
Method	Value	90% CI		
Noncentrality parameter (NCP)	6.0	0.0	19.9	
Minimum value of the discrepancy (FMIN)	0.08	0.0	0.14	
Root mean squared error of approximation (RMSEA)	0.08	0.0	0.15	
Except for a constant scale factor (ECVI)	0.30	0.25	0.39	
Method	Value	DF	*p*-value	CMIN/DF
Minimized value of the discrepancy function (CMIN)	12.0	6	0.06	2.0

Tests of the goodness of fit used. All figures are shown for the default model fit

## Discussion

We were able to translate, adapt, and validate the SCHNOS into French, producing the F-SCHNOS. This French version was shown to be conceptually and psychometrically equivalent to the original English version. The meticulous process of translation and cultural adaptation is supported by many international guidelines [4, 5, 7].

This multistep procedure is of paramount importance is order to achieve not only semantic equivalence, but to ensure that the original content and concepts are respected and adapted to the population which the instrument targets.

The F-SCHNOS is a reliable instrument, as demonstrated by a high internal consistency for both SCHNOS-O and SCHNOS-C. These results are very similar to the Cronbach’s alpha of the original English version [2]. It is also a valid instrument, which refers to its ability to measure accurately the outcome of interest. Multiple analyses are required in order to prove validity [8], as it has already been demonstrated for the original English version of the SCHNOS [9]. The positive and significant correlation between each item of the F-SCHNOS-O and F-SCHNOS-C is a demonstration. The methodology used for the translation process is also a safeguard of content validity.

In the test-retest phase of the study, we demonstrated the reproducibility of our instrument. The participants in this study answered in a positively correlated manner between the 2 weeks interval for both SCHNOS-O and SCHNOS-C. Furthermore, for the SCHNOS-C, their answers were not significantly different in the 2 weeks interval, which highlight the proper reproducibility of our instrument. There was although a significant difference for the SCHNOS-C in the Wilcoxon signed-rank test. We think that the method of administration of the questionnaire might have had an influence over this result. Since aesthetic appearance of the nose is a more sensitive subject for many patients, they might have been more reluctant to answer sincerely by phone at the 2 weeks interval than at the first consultation, were the questionnaire was filled in writing.

The small number of participants recruited for the psychometric validation is a limitation to this study. However, many similar translation studies achieved a validation process with similar or a more limited number of participants [10–13]. Furthermore, a small sample size would only diminish the chances of finding a significant association. Since our results showed the reliability and validity of the French SCHNOS, especially with IRT, we believe that the associations would only be stronger with a larger sample size.

This study is the first one to generate a French-language version of the SCHNOS questionnaire. Such adapted questionnaires are important in health-related quality of life evaluation. They are useful for screening and monitoring the individual patient and in the evaluation of health outcomes [14]; but also providing comparable results for international research [4].

## Conclusions

In conclusion, we successfully translated, adapted, and validated the SCHNOS into French in order to help with the evaluation of functional and cosmetic outcomes of rhinoplasty patients. We hope that this will provide an additional tool to the clinician who is evaluation the French-speaking rhinoplasty patient.

## Abbreviations

CFA: Confirmatory factor analysis
IRT: Item Reponse Theory
ISPOR: International Society for Pharmacoeconomics and Outcomes Research
PROM: Patient-reported outcome measure
RMS: Rating Scale Model
RMSEA: Root mean square error of approximation
SCHNOS: Standardized Cosmesis and Health Nasal Outcome Survey
SCHNOS-C: Standardized Cosmesis and Health Nasal Outcome Survey – Cosmesis domain
SCHNOS-O: Standardized Cosmesis and Health Nasal Outcome Survey: Obstruction domain
